# Correlation between intraoperative and postoperative vaulting of the EVO implantable Collamer lens: a retrospective study of real-time observations of vaulting using the RESCAN 700 system

**DOI:** 10.1186/s12886-021-02237-2

**Published:** 2022-01-03

**Authors:** Nian Guan, Xiao-Nong Zhang, Wan-Jun Zhang

**Affiliations:** 1 Department of Refractive, Wuhan Bright Eye Hospital, Wuhan, 430000 Hubei China; 2 Department of Refractive, Hefei Bright Eye Hospital, Hefei, 230000 Anhui China

**Keywords:** Lenses, intraocular, lens implantation, intraocular, Tomography, optical coherence, Refractive surgery, Post-operative vault

## Abstract

**Background:**

Implantable Collamer lens (ICL) vaulting is one of the most important parameters for the safety, aqueous humor circulation, and lens transparency after ICL implantation. This study aimed to investigate the factors associated with the actual vaulting after refractive EVO-ICL surgery.

**Methods:**

This retrospective study included patients who underwent EVO-ICL surgery at a tertiary eye hospital between October and December 2019. A RESCAN 700 was used for the intraoperative and CIRRUS HD-OCT was used for postoperative observation of vaulting. Subjective and objective refractions, anterior ocular segment, corneal morphology, intraocular pressure (IOP), anterior chamber volume (ACV), crystalline lens rise (CLR), white-to-white distance (WTW), anterior chamber depth (ACD), axial length, corneal endothelial cell density (ECD), and fundoscopy were examined. A multivariable analysis was performed to determine the factors independently associated with 1-month postoperative vaulting.

**Results:**

Fifty-one patients (102 eyes) were included. Compared with the eyes with normal vaulting, those with high vaulting had higher preoperative diopter values (*P* = 0.039), lower preoperative corrected visual acuity (*P* = 0.006), lower preoperative IOP (*P* = 0.029), higher preoperative ACD (*P* = 0.004), lower preoperative CLR (*P* = 0.046), higher ICL spherical equivalent (*P* = 0.030), higher intraoperative vaulting (*P* < 0.001), and lower IOP at 1 month (*P* = 0.045). The multivariable analysis showed that the only factor independently associated with high vaulting at 1 month after surgery was the intraoperative vaulting value (odds ratio = 1.005, 95% confidence interval: 1.002–1.007, *P* < 0.001). The intraoperative and 1-month postoperative vaulting values were positively correlated (R^2^ = 0.562).

**Conclusions:**

The RESCAN700 system can be used to perform intraoperative optical coherence tomography to predict the vaulting value of ICL at 1 month.

## Background

Refractive errors of the eye are common conditions and include myopia, hyperopia, and astigmatism. The worldwide prevalence of myopia is 1.45 billion [1], while hyperopia and astigmatism are found in 30.9 and 40.4% of adults [2]. Such errors arise when the images are not sharply focused on the retina due to the eyeball length and shape of the cornea. Corrective glasses or contact lenses are the most common methods used to achieve better vision.

Implantable Collamer lens (ICL) is another option for the correction of refractive errors. The Visian ICL™ (STAAR Surgical, Nidau, Switzerland) is a posterior chamber phakic intraocular lens (IOL) [3–5]. The Visian ICL is available in the V4c, EVO, EVO+ V5, and Toric versions [6–9]. The Visian ICL is indicated for adults of 21–45 years of age to correct myopia of − 3.0 of − 15.0 D with < 2.5 D of astigmatism or to reduce myopia of − 15.0 to − 20.0 D with < 2.5 D of astigmatism, the anterior chamber depth (ACD) has to be at least 3.0 mm, and refractive history has to be stable (within 0.5 D) for at least 1 year [10, 11]. The Visian Toric ICL is indicated in patients of 21–45 years of age to correct myopic astigmatism of spherical − 3.0 to − 15.0 D and cylinder 1.0 to 4.0 D or to reduce myopic astigmatism of spherical − 15.0 to − 20.0 D and cylinder 1.0 to 4.0 D, the ACD has to be at least 3.0 mm, and refractive history has to be stable (within 0.5 D) for at least 1 year [10, 11]. For both types, the contraindications are anterior chamber angle less than grade II, pregnancy, nursing, low epithelial cell density (ECD) (< 1900–3875 cells/mm^2^, depending upon age) [10, 11]. The EVO-ICL is based on an artificial hole and achieves acceptable safety [12] and is similar to traditional ICLs in terms of high-order aberrations and contrast sensitivity [13]. Nevertheless, the most challenging parameter in ICL implantation is the accurate prediction of vaulting, and precise and optimal vaulting is the key parameter for successful ICL implantation [14]. An improper vaulting can lead to adverse events such as pupillary block, iris touch, angle-closure glaucoma, anterior lens opacification, and early cataract [15–17]. About 2.6% of implanted ICLs have improper vaulting and require exchange [18–22].

Previous methods for determining vaulting include white-to-white diameter (manually or with imaging systems) or sulcus-to-sulcus diameter using high-frequency ultrasound [23, 24]. Later, optical coherence tomography (OCT) was added to refine the prediction [6, 25, 26]. Recent OCT systems built within the operating microscope now allow for more precise eye surgeries and for various measurements during surgery [27–29]. Nevertheless, only one study examined the use of intraoperative OCT to determine ICL vaulting [30]. A study showed that external light continuously affects vaulting value [31]. Therefore, intraoperative measurements in a controlled environment such as the microscope might be more precise to determine ICL vaulting [30], but confirmation is needed. Of note, a recent multivariable model explains only 34% of the variability of lens vaulting among individuals [32], indicating that studies are still necessary to determine the factors influencing vaulting and allow a more precise determination of ICL vaulting. Hence, additional studies are necessary to refine the prediction of ICL vaulting.

This study aimed to investigate the factors associated with the actual vaulting after refractive EVO-ICL surgery and the correlation between intraoperative vaulting and the actual vaulting at 1 month after surgery to determine whether OCT device during surgery could provide some clinical help. The results might help a better prediction of ICL vaulting and avoid the need for early ICL exchange.

## Methods

### Study design and patients

This retrospective study included patients who underwent EVO-ICL surgery at a tertiary eye hospital between October and December 2019. This study was approved by the Ethics Committee of the Hospital (approval code: 2019[02]). The present study was also registered at http://www.chictr.org.cn (registration number: ChiCTR2000032226). This study was conducted according to the tenets of the Declaration of Helsinki and the Good Clinical Practice. The requirement for individual informed consent was waived by the committee because of the retrospective nature of the study. All patients provided consent for intraocular treatments.

The inclusion criteria were 1) 21–45 years of age, 2) ACD > 2.8 mm, 3) corneal ECD > 2000/mm^2^, and 4) completed EVO-ICL surgery and follow-up in this hospital. The exclusion criteria were 1) other eye diseases that caused a visual loss, such as cataracts and glaucoma, 2) systemic diseases such as diabetes, autoimmune diseases, or collagen diseases that could affect postoperative healing, or 3) being unable to measure vaulting due to unclear intraoperative OCT images.

### Preoperative measurements

All data were extracted from the electronic medical charts of the patients. As per routine preoperative management at the authors’ center during the study period, the preoperative diopter of the correcting lenses, corrected visual acuity (CVA), white-to-white distance (WTW), intraocular pressure (IOP), ACD, anterior chamber volume (ACV), crystalline lens rise (CLR), axial length, and ECD were recorded in the charts. During the study period, the RESCAN 700 system (Carl Zeiss GmbH, Oberkochen, Germany) was used intraoperatively to measure EVO-ICL vaulting. Uncorrected visual acuity (UCVA) and best-corrected visual acuity (BCVA) were checked using an international standard visual acuity chart (converted into logMAR visual acuity).

During the study period, a CV-5000 comprehensive refractometer (Topcon Corporation, Tokyo, Japan) was used to measure subjective and objective refractions. An SL-115 Classic slit lamp microscope (Carl Zeiss GmbH, Oberkochen, Germany) was used to determine the anterior ocular segment. A Pentacam HR three-dimensional panoramic analyzer for the anterior segment (Oculus, Wetzlar, Germany) was used to check corneal morphology, ACV, CLR, and WTW. A CT-800 non-contact tonometer (Topcon Corporation, Tokyo, Japan) was used to measure IOP. An IOL Master 700 biometer (Carl Zeiss GmbH, Oberkochen, Germany) was used to measure ACD and axial length. An SP-3000P corneal endothelial cell counter (Topcon Corporation, Tokyo, Japan) was used to measure corneal ECD. A V90C non-contact slit lamp pre-set lens (Halma plc, Amersham, UK) was used to perform fundoscopy. A RESCAN 700 (Carl Zeiss AG, Oberkochen, Germany) was used for intraoperative imaging, SD-OCT was used for scan imaging, and the ImageJ software was used to measure the ICL vault value. A CIRRUS HD-OCT (Carl Zeiss GmbH, Oberkochen, Germany) was used to measure the postoperative distance between the posterior surface of the EVO-ICL and the anterior lens capsule, namely the vault value. All measurements were performed by an ophthalmologist.

### EVO-ICL surgery

All procedures were performed by the same ophthalmologist. The size of the EVO-ICL was determined based on WTW, ACD, ACV, and CLR. The manufacturer’s online system was used to calculate the EVO-ICL diopter (STAAR Surgical Co., Monrovia, CA, USA). At 3 days before surgery, levofloxacin eye drops (Santen Pharmaceutical Co., Ltd., Osaka, Japan) were continuously administrated 4 times/day. At 30 min before surgery, compound tropicamide eye drops (Santen Pharmaceutical Co., Ltd., Osaka, Japan) were used for mydriasis. Oxybuprocaine hydrochloride eye drops (Santen Pharmaceutical Co., Ltd., Osaka, Japan) were used to perform surface anesthesia. The axis of corneal astigmatism was marked under the slit lamp before surgery. Conventional disinfection and draping were conducted. The conjunctival sac was washed. The main incision was made at the steepest meridian of the cornea. A syringe was used to inject the EVO-ICL into the anterior chamber. An appropriate amount of 15 mg/ml medical sodium hyaluronate gel (Hangzhou Singclean Medical Products Co., Ltd., Hangzhou, China) was injected above the EVO-ICL to maintain the ACD. The four angles of the EVO-ICL were adjusted to the ciliary sulcus behind the iris with the adjustment hook, and the EVO-ICL was adjusted to the marked area and the residual viscoelastic in the anterior chamber. An Icare rebound tonometer (Icare Finland Oy, Vantaa, Finland) was used to measure the IOP, controlled at 15–18 mmHg by replenishing and releasing aqueous humor. A RESCAN 700 microscope (Carl Zeiss AG, Oberkochen, Germany) was used to perform the SD-OCT scan imaging. The five-line scanning mode was used, with a scanning depth of 2.0 mm and a scanning length of 2.0 mm. The distance between the posterior surface of the EVO-ICL and anterior lens capsule was observed, and the snapshot mode was used to save the screenshot after clearing. After the end of the surgery, tobramycin dexamethasone eye drops (Alcon-Couvreur SA, Puurs, Belgium) were used.

### Intraoperative measurement of vaulting

A RESCAN 700 (Carl Zeiss AG, Oberkochen, Germany) was used for intraoperative imaging, and SD-OCT was used for scanning imaging. For intraoperative SD-OCT image export, the ImageJ software (version 1.48) was used for processing, and the scanning depth was adjusted to 2.0 mm. The distance between the posterior surface of EVO-ICL and the anterior lens capsule was measured. All measurements were conducted three times, and the average values were recorded, namely the intraoperative vaulting values.

### Follow-up

The 1-month follow-up data were extracted from the charts. During follow-up, the distance between the posterior surface of the EVO-ICL and anterior lens capsule (namely, the vaulting value) was measured using a CIRRUS HD-OCT (Carl Zeiss AG, Oberkochen, Germany). Under the same indoor light for all patients, all measurements were performed by the same ophthalmologist three times, and the average values were recorded. For the vault at 1 month after surgery, 250–750 μm was defined as normal vaulting, < 250 μm as low vaulting, and > 750 μm as high vaulting [33, 34]. At the same time, visual acuity, IOP, and diopter were measured.

### Statistical analysis

SPSS 22.0 (IBM Corp., Armonk, NY, USA) was used for data processing and statistical analyses. Normally distributed continuous data (according to the Kolmogorov-Smirnov test) were presented as means ± standard deviations and analyzed using Student’s t-test. Non-normally distributed data were presented as medians (ranges) and analyzed using the Mann-Whitney U-test. Categorical data were presented as frequencies (percentage) and analyzed using the chi-square test or Fisher’s exact test. For the multivariable analysis, high vaulting at 1 month after surgery was used as the dependent variable, and the factors with significant between-group differences (*P* < 0.05) in the univariable analyses (enter method) were used as the independent variables. Binary logistic regression analysis was performed. Linear correlation analysis was performed regarding the intraoperative and postoperative vaulting. Two-sided (except for the chi-square test) *P*-values < 0.05 were considered statistically significant.

## Results

### Characteristics of the patients

**Fig. 1:**
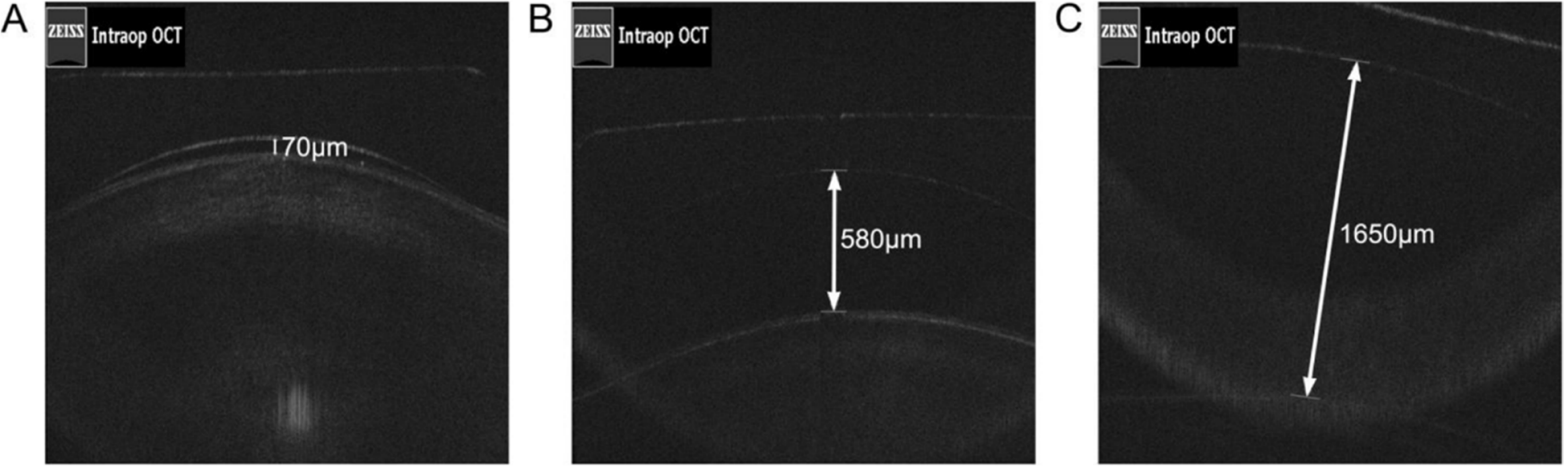
Typical figures for measuring vaulting (intraoperative). **A** Low intraoperative vaulting; **B** Normal intraoperative vaulting; **C** High intraoperative vaulting

A total of 56 patients (112 eyes) were eligible. Among them, vaulting could not be measured in five patients (10 eyes) by intraoperative OCT due to unclear intraoperative OCT images. Finally, 51 patients (102 eyes) were included. There were two (2.0%) eyes with low vaulting postoperatively, and two (2.0%) eyes underwent lens exchange due to high vaulting. Figure 1 presents typical vaulting measurements. Given that there were only two patients with low vaulting, this study analyzed patients with normal and high vaulting.

Table 1 presents the characteristics of the patients. Compared with the eyes with normal vaulting, those with high vaulting had higher preoperative diopter values (− 8.8 (− 18,-5) vs. -8.4 (− 18,-2.8) D, *P* = 0.039), lower preoperative CVA (0 (0,0.4) vs. 0 (0,0.5) LogMAR, *P* = 0.006), lower preoperative IOP (17 (14,21) vs. 19 (13,22) mmHg, *P* = 0.029), higher preoperative ACD (3.4 (2.8,3.7) vs. 3.1 (2.8,3.7) mm, *P* = 0.004), lower preoperative CLR (190 (0,390) vs. 230 (0,520) μm, *P* = 0.046), higher ICL spherical equivalent (SE) (− 10 (− 18,-5.5) vs. -9.5 (− 18,-3.5) D, *P* = 0.030), higher intraoperative vaulting (1001.2 ± 284.8 vs. 657.2 ± 279.3 μm, *P* < 0.001), and lower IOP at 1 month (17 (14,21) vs. 18 (13,23) mmHg, *P* = 0.045).

**Table 1:** Characteristics of the patients

Characteristics	All (*n* = 100)	Normal vaulting (*n* = 67)	High vaulting (*n* = 33)	*P*
Sex (male), n (%)	35 (35)	26 (38.8)	9 (27.3)	0.256
Age (years), median (range)	25.5 (21,40)	26 (21,40)	25 (21,39)	0.680
Preoperative diopter SE (D), median (range)	−8.5 (− 18,-2.8)	−8.4 (− 18,-2.8)	−8.8 (− 18,-5)	0.039
Preoperative CVA (LogMar), median (range)	0 (0,0.5)	0 (0,0.5)	0 (0,0.4)	0.006
Preoperative IOP (mmHg), median (range)	18 (13,22)	19 (13,22)	17 (14,21)	0.029
Preoperative ACD (mm), median (range)	3.2 (2.8,3.7)	3.1 (2.8,3.7)	3.4 (2.8,3.7)	0.004
Preoperative ACV (μL), median (range)	205 (128,562)	204 (131,562)	217 (128,307)	0.172
Preoperative axial length (mm), mean ± SD	26.8 ± 1.3	26.7 ± 1.3	27.1 ± 1.1	0.080
Preoperative corneal ECD, mean ± SD	2914.1 ± 233.7	2910.3 ± 263.2	2922 ± 161.2	0.785
Preoperative WTW (mm), median (range)	11.7 (10.7,12.6)	11.6 (10.7,12.5)	11.8 (10.8,12.6)	0.091
Preoperative CLR (μm), median (range)	210 (0,520)	230 (0,520)	190 (0,390)	0.046
ICL size (mm), n (%)				0.175
121	4 (4)	3 (4.5)	1 (3)	
126	38 (38)	30 (44.8)	8 (24.2)	
132	52 (52)	30 (44.8)	22 (66.7)	
137	6 (6)	4 (6)	2 (6.1)	
ICL degree SE (D), median (range)	−9.5 (−18,-3.5)	−9.5 (−18,-3.5)	−10 (− 18,-5.5)	0.030
Intraoperative vaulting (μm), mean ± SD	770.7 ± 323.5	657.2 ± 279.3	1001.2 ± 284.8	< 0.001
Vaulting at 1 month after surgery (μm), median (range)	634 (252,1650)	560 (252,730)	910 (760,1650)	< 0.001
Visual acuity at 1 month after surgery (LogMar), median (range)	−0.1 (− 0.2,0.4)	−0.1 (− 0.2,0.4)	−0.1 (− 0.2,0.2)	0.284
Diopter SE at 1 month after surgery (D), median (range)	0.3 (−1.5,1.3)	0.3 (−1.5,1.3)	0.3 (− 0.3,1)	0.127
IOP (mmHg) at 1 month after surgery, median (range)	18 (13,23)	18 (13,23)	17 (14,21)	0.045

Spherical equivalent = sphere power plus 1/2 cylinder power

*SE* Spherical equivalent, *CVA* Corrected visual acuity, *IOP* Intraocular pressure, *ACD* Anterior chamber depth, *ACV* Anterior chamber volume, *ECD* Endothelial cell density, *WTW* White-to-white distance, *CLR* Crystalline lens rise

### Multivariable analysis

Based on the univariable analyses, preoperative diopter (OR = 0.846, 95%CI:0.73–0.982, *P* = 0.028), preoperative CVA (OR = 210.273, 95%CI: 1.444–30,626.133, *P* = 0.035), preoperative IOP (OR = 0.825, 95%CI: 0.683–0.998, *P* = 0.048), preoperative ACD (OR = 12.695, 95%CI:1.999–80.604, *P* = 0.007), preoperative CLR (OR = 0.996, 95%CI:0.992–0.999, *P* = 0.022), ICL degree SE (OR = 0.837, 95%CI: 0.718–0.975, *P* = 0.023), and intraoperative vaulting (OR = 1.004, 95%CI:1.002–1.006, *P* < 0.001) were entered the multivariable analysis (Table 2). The multivariable analysis showed that the only factor independently associated with high vaulting at 1 month after surgery was the intraoperative vaulting value (OR = 1.005, 95%CI: 1.002,1.007, *P* < 0.001) (Table 2).

**Table 2:** Independent influencing factors of high vaulting at 1 month after the operation

Characteristics	Univariable analysis			Multivariable analysis		
	OR	95%CI	*P*	OR	95%CI	*P*
Sex (male)	0.591	0.238,1.469	0.258			
Age (years)	0.976	0.889,1.071	0.602			
Preoperative diopter SE (D)	0.846	0.73,0.982	0.028	1.038	0.18,5.985	0.966
Preoperative CVA (LogMar)	210.273	1.444,30,626.133	0.035	1.374	0.001,3409.882	0.937
Preoperative IOP (mmHg)	0.825	0.683,0.998	0.048	0.849	0.659,1.093	0.205
Preoperative ACD (mm)	12.695	1.999,80.604	0.007	5.955	0.415,85.456	0.189
Preoperative ACV (μL)	1.002	0.993,1.011	0.673			
Preoperative axial length (mm)	1.362	0.96,1.933	0.084			
Preoperative corneal ECD	1.000	0.998,1.002	0.813			
Preoperative WTW (mm)	2.050	0.741,5.674	0.167			
Preoperative CLR (μm)	0.996	0.992,0.999	0.022	0.997	0.992,1.002	0.274
ICL size (mm)						
12.1	Ref			Ref		
12.6	0.800	0.073,8.764	0.855			
13.2	2.200	0.214,22.591	0.507			
13.7	1.500	0.089,25.392	0.779			
ICL degree SE (D)	0.837	0.718,0.975	0.023	0.730	0.129,4.138	0.723
Intraoperative vault (μm)	1.004	1.002,1.006	< 0.001	1.005	1.002,1.007	< 0.001

*OR* Odds ratio, *CI* Confidence interval, SE Spherical equivalent, *CVA* Corrected visual acuity, *IOP* Intraocular pressure, *ACD* Anterior chamber depth, *ACV* Anterior chamber volume, *ECD* Endothelial cell density, *WTW* White-to-white distance, *CLR* Crystalline lens rise

### Correlation analysis

**Fig. 2:**
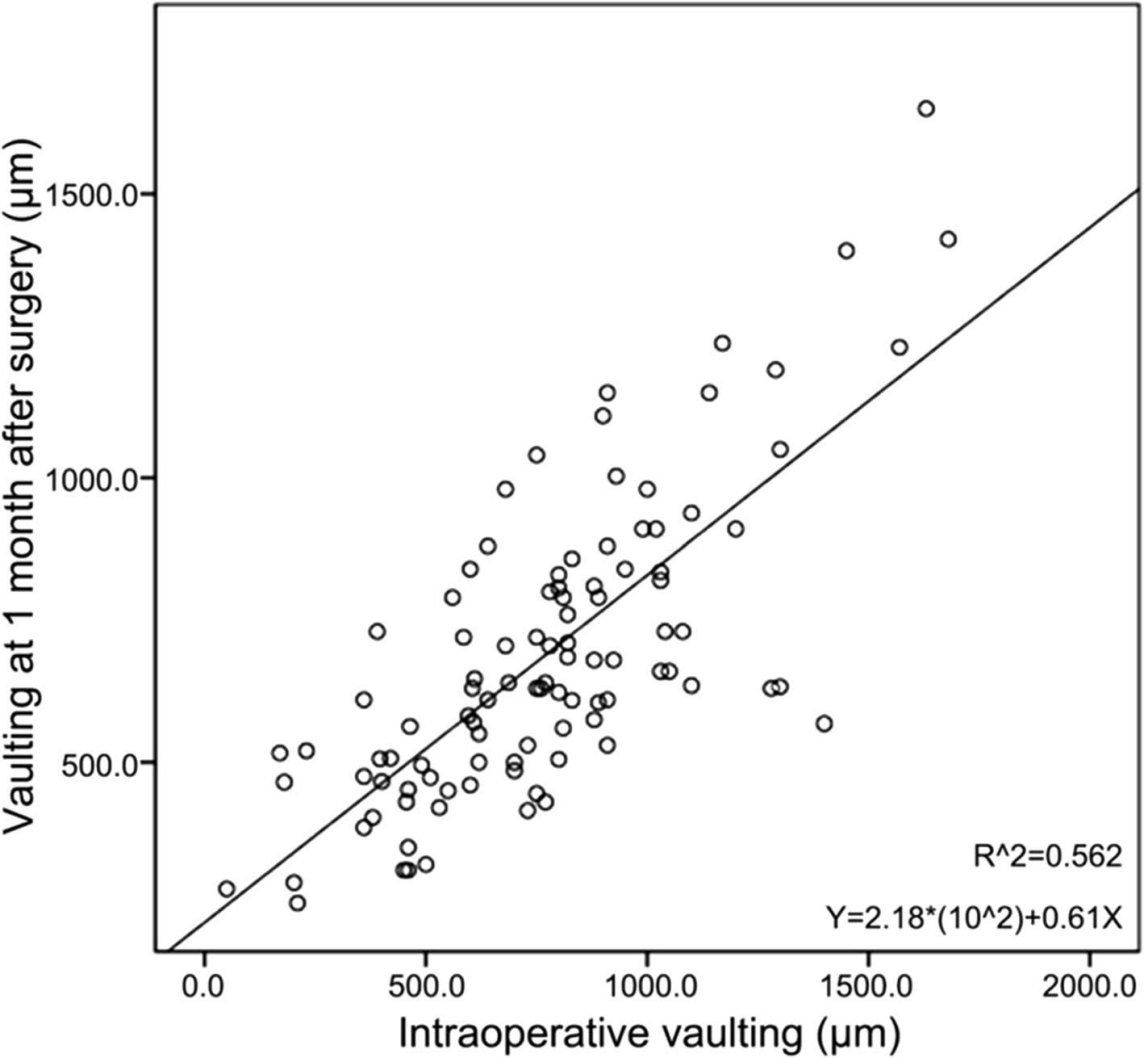
Linear correlation between intraoperative vaulting and the vaulting at 1 month after surgery. X-axis: intraoperative vaulting. Y-axis: vaulting at 1 month after surgery

The results of the linear correlation analysis of intraoperative and postoperative vaulting are shown in Fig. 2. The correlation was positive and significant (R^2^ = 0.562).

## Discussion

With the improvements in IOLs, IOL implantation is considered a safe procedure [14]. Still, ICL vaulting is one of the most important parameters for ICL implantation because improper vaulting can result in complications, reintervention, and low patient satisfaction. Even with the best WTW, STS, and ACD measurements, improper vaulting occurs, and reintervention is necessary for about 2.6% of the patients [18, 20–22, 35]. Thus, predicting postoperative vaulting has clinical value. This study aimed to investigate the factors associated with actual vaulting after EVO-ICL implantation and the correlation between intraoperative and 1-month vaulting using OCT. The results suggest that the RESCAN700 system can be used to perform intraoperative OCT to predict the vaulting value at 1 month. Taking into account the positive correlation between intraoperative and 1-month vaulting, optimization interventions could be carried out in time to obtain better results when abnormal intraoperative vaulting is observed.

The RESCAN 700 is the latest generation of operating microscopes integrating the LUEMRA microscope platform and OCT. It can be used to perform real-time observations of OCT images during surgery. Studies reported using the RESCAN 700 system in vitreoretinal surgery, corneal transplantation, and cataract surgery [36–40]. Only one recent study used the RESCAN 700 to examine the vaulting after ICL implantation [30]. The accuracy of real-time intraoperative measurement of vaulting is critical for the success of ICL implantation. Indeed, the implantation of an ICL with the correct vaulting from the start will avoid complications (mechanical contact with the lens, pupillary block, iris touch, angle-closure glaucoma, anterior lens opacification, and early cataract [15–17]) and the need for reoperation and lens exchange [41]. This precision would save healthcare resources and money.

The traditional methods to determine vaulting based on WTW and ACD lead to about 20% of the patients being outside the accepted vaulting range after surgery [35, 42]. The STS can also be used, but the relationship between the WTW and STS is affected by myopia’s degree [43–48]. OCT is a valuable tool for predicting vaulting [6, 25, 26, 30]. In the present study, two eyes had too high vaulting, and two eyes had too low vaulting, leading to 4% of the eyes being outside the appropriate vaulting range. In addition, a 90-μm difference was observed between the intraoperative and 1-month values, similar to the 100-μm difference observed by Torbey et al. [30], but Titiyal et al. [49] observed a difference of only 7 μm between the intraoperative and 3-month postoperative vaulting values. These differences are likely due to the surgery itself, the use of irrigation, intraoperative adjustment in IOP, and the use of drugs to dilate the pupil, while the OCT at 1-month was measured on a physiological pupil. Indeed, vaulting is affected by pupil size [50]. In addition, Titiyal et al. [49] used measurement methods that resulted in distorted images and without using ICL thickness as a reference to evaluate distortion. Despite this difference, the intraoperative and 1-month vaulting values were highly correlated, as supported by Torbey et al. [30]. On the other hand, a study showed only a 7-μm difference between the intraoperative and 3-month vaulting values [49]. The wide-angle OCT image acquisition is associated with image distortion and could be a source of bias. In addition, the previous study [49] did not mention if a miotic agent was used before measurement.

Because of this difference in vaulting, it is difficult to determine whether an ICL should be exchanged when observing limit values. Nevertheless, as suggested by Torbey et al. [30], the ICL should be exchanged within the same operative session in the presence of extreme vaulting values. Doing so will avoid a second surgery, improving safety and patient satisfaction. Furthermore, a good intraoperative vault value in one eye is conducive for higher confidence for the fellow eye when performing bilateral ICL implantations. Trancon et al. [32] elaborated a multivariable model that could predict vaulting and explain 34% of the variance in vaulting values; lens diameter, horizontal anterior chamber angle distance, CLR, ICL spherical equivalent, and patient age were independently associated with vaulting. In the present study, only intraoperative vaulting was associated with the value at 1 month. This discrepancy could be due to the number of eyes, different OCT systems, and different drugs used during surgery. Two eyes had ICL with too high vaulting and the ICL had to be exchanged in order to prevent short- and long-term complications [15–17]. The rate of 2% reported here is within the numbers reported by the literature [18–22]. The two eyes with low vaulting were not re-operated, but close follow-up was performed. The low vaulting observed in the two eyes might be due to the smaller pupil diameters, as suggested by a previous study [51].

ICL vaulting decreases with age because of the increased crystalline lens thickness with age, and ICL vaulting in patients with myopia decreases over 12 years in one study [52]. Previous studies showed that high vaulting could lead to a small anterior chamber (AC) angle and increased IOP [16, 53–55], but increased IOP was not observed in the present study, possibly because of the small sample size. Despite that the IOP changes were reported to remain within normal physiological values [16, 53–55], these changes could contribute to glaucoma and other complications. The AC angle was not measured in this research. Further research is needed to determine the relationship between AC angle and ICL vaulting. The univariable analysis also showed that the preoperative diopter was associated with high postoperative vaulting. However, preoperative diopter was not independently associated with high vaulting in the multivariable analysis, suggesting that the difference reflected in Table 1 are interfered with by various other factors. In the selection of ICL size during surgery, the preoperative WTW index was taken into account when using the manufacturer’s online tool for ICL selection. The WTW was also included in the univariable analysis because of that. Still, the analysis showed that preoperative WTW had little relationship with postoperative vaulting, which was similar to the previous research [56].

The strengths of this study reside in the current lack of data about the intraoperative measurement of vaulting, which has a direct influence on clinical practice and the safety of ICL. In addition, the previous study by Titiyal et al. [49] included 26 patients (45 eyes), while the present study included 51 patients (102 eyes). This study also has limitations. The study was performed at a single hospital, and the number of included eyes was small. Because of the study’s retrospective nature, only the routine follow-up at 1 month was available, and the changes in vaulting over time could not be examined. In fact, the study period (October–November 2019) was just before the COVID-19 outbreak and nationwide lockdown, preventing the patients from having additional follow-up. Only one type of ICL was examined, and different ICL architectures might affect vaulting and postoperative outcomes. Future studies should include more patients and should be prospective to include more follow-up time points and longer follow-up.

## Conclusion

The RESCAN700 system can be used to perform intraoperative OCT to predict the vaulting value at 1 month. Therefore, the vaulting value observed intraoperatively is probably predictive of the actual postoperative vaulting value. It could allow immediate replacement of the IOL in case of abnormal intraoperative vaulting, resulting in better surgical outcomes.

## Data Availability

The datasets used and/or analyzed during the current study are available from the corresponding author on reasonable request.

## Abbreviations

ACD: Anterior chamber depth
ACV: Anterior chamber volume
AS-OCT: Anterior segment optical coherence tomography
BCVA: Best-corrected visual acuity
CLR: Crystalline lens rise
CVA: Corrected visual acuity
ECD: Endothelial cell density
ICL: Implantable Collamer lens
IOL: Intraocular lens
OCT: Optical coherence tomography
SE: Spherical equivalent
UCVA: Uncorrected visual acuity
WTW: White-to-white distance
