# Correction to: Correlation between intraoperative and postoperative vaulting of the EVO implantable Collamer lens: a retrospective study of real-time observations of vaulting using the RESCAN 700 system

**DOI:** 10.1186/s12886-022-02259-4

**Published:** 2022-02-23

**Authors:** Nian Guan, Xiao-Nong Zhang, Wan-Jun Zhang

**Affiliations:** 1 Department of Refractive, Wuhan Bright Eye Hospital, Wuhan, 430000 Hubei China; 2 Department of Refractive, Hefei Bright Eye Hospital, Hefei, 230000 Anhui China

**Correction to: BMC Ophthalmol 22, 2 (2021)**

**https://doi.org/10.1186/s12886-021-02237-2**

Following the publication of the original article [1], we were notified that Figures 1 and 2 were incorrect.

Originally published figures:

**Figure 1.**
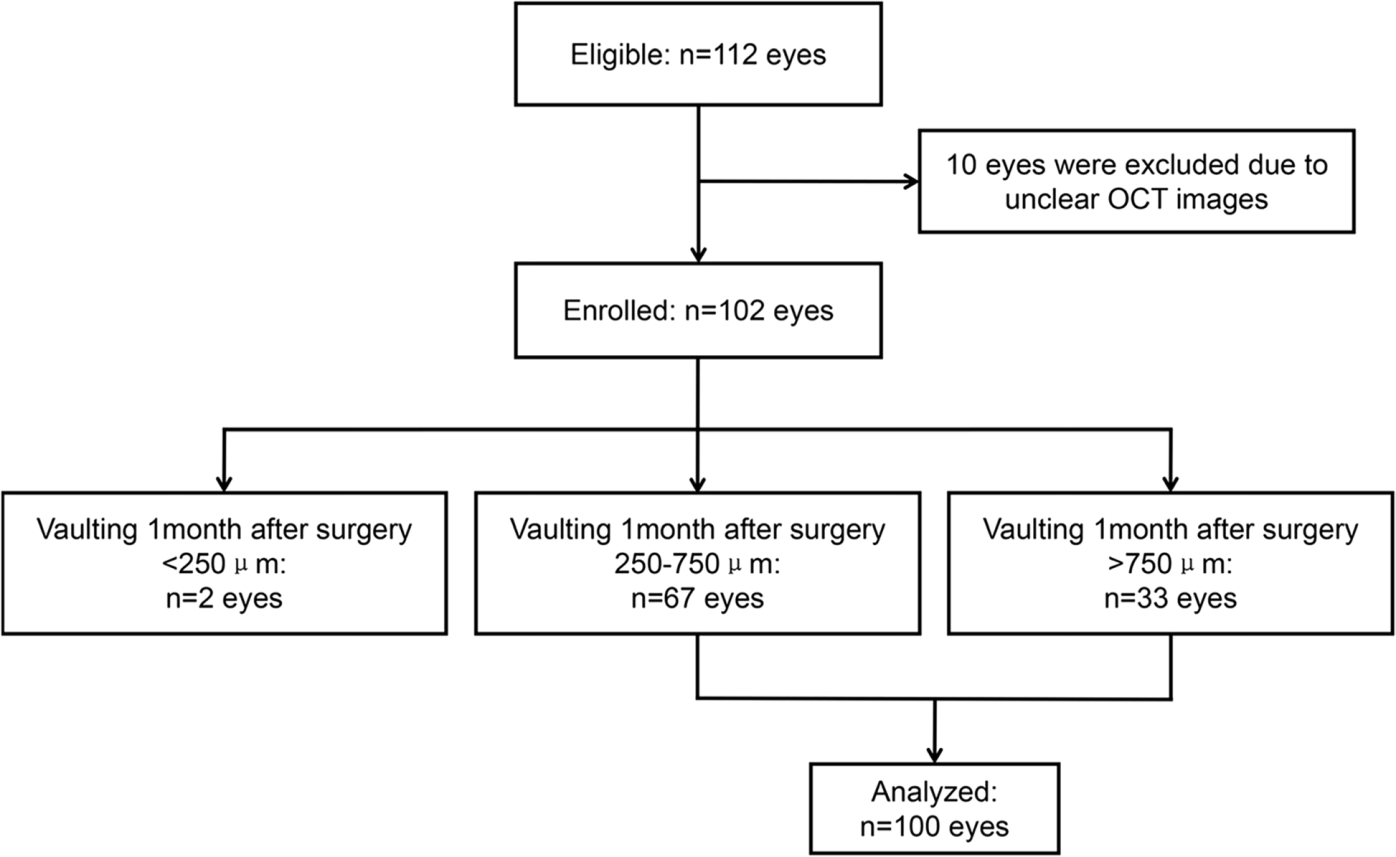
Linear correlation between intraoperative vaulting and the vaulting at 1 month after surgery. X-axis: intraoperative vaulting. Y-axis: vaulting at 1 month after surgery.

**Fig. 2:**
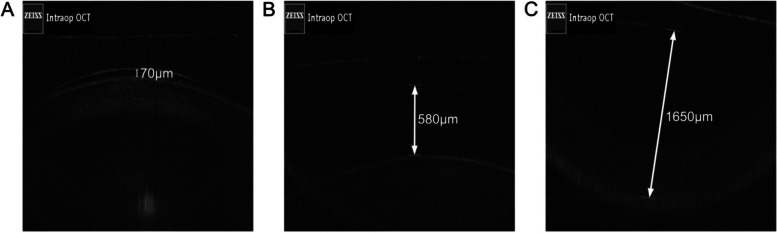
Typical figures for measuring vaulting (intraoperative). (A) Low intraoperative vaulting; (B) Normal intraoperative vaulting; (C) High intraoperative vaulting.

Corrected figures:

**Fig. 1:**
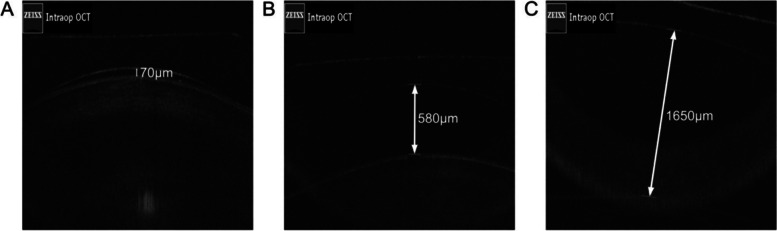
Typical figures for measuring vaulting (intraoperative). (A) Low intraoperative vaulting; (B) Normal intraoperative vaulting; (C) High intraoperative vaulting.

**Figure 2.**
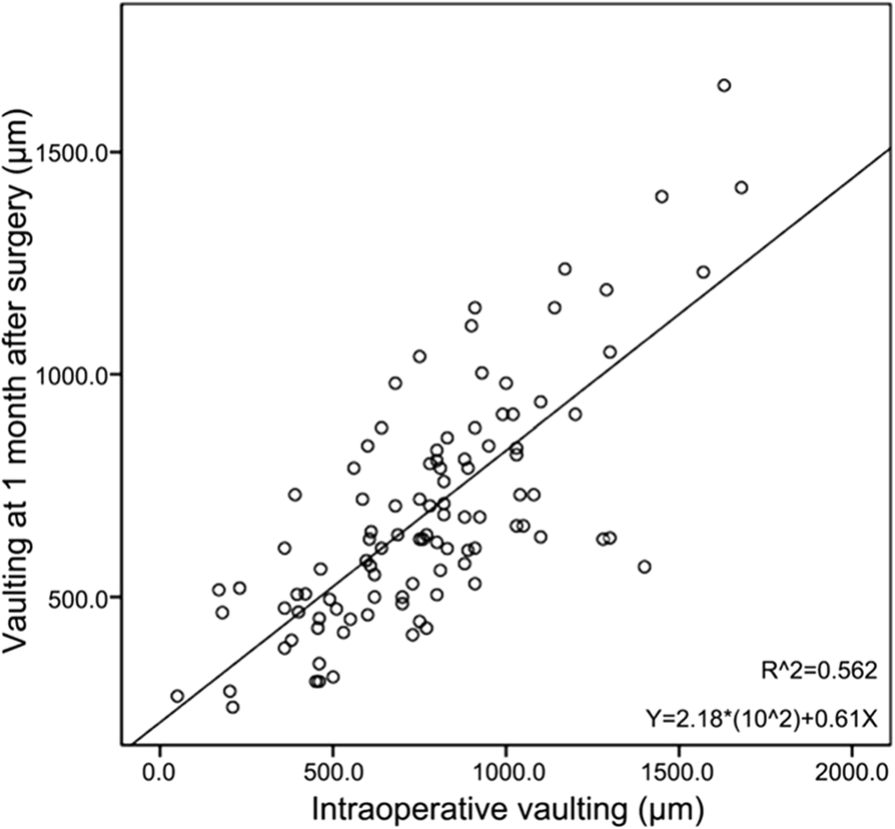
Linear correlation between intraoperative vaulting and the vaulting at 1 month after surgery. X-axis: intraoperative vaulting. Y-axis: vaulting at 1 month after surgery.

The original article has been corrected.
